# Inflammatory Signatures of Pathogenic and Non-Pathogenic Leptospira Infection in Susceptible C3H-HeJ Mice

**DOI:** 10.3389/fcimb.2021.677999

**Published:** 2021-06-24

**Authors:** Advait Shetty, Suman Kundu, Maria Gomes-Solecki

**Affiliations:** ^1^ Department of Pharmaceutical Sciences, The University of Tennessee Health Science Center, Memphis, TN, United States; ^2^ Department of Microbiology, Immunology and Biochemistry, The University of Tennessee Health Science Center, Memphis, TN, United States

**Keywords:** immune cells, flow cytometry, proteome array, cytokines, chemokines, *L. biflexa*, *L. interrogans*, Leptospirosis

## Abstract

The exact global impact of leptospirosis is unknown due to inadequate surveillance systems in place in most low-income countries. In this study, we analyzed the differences in mouse inflammatory signatures involved in pathogenic versus non-pathogenic Leptospira recognition at 24h and 72h post infection. Injection of C3H-HeJ mice with non-pathogenic *L. biflexa* increased circulation of a few chemokines (5/21, 24%) without secretion of cytokines in blood that resulted in engagement of resident macrophages, dendritic cells, neutrophils and NK cells without engagement of T cells. In contrast, pathogenic *L. interrogans* induced circulation of a much higher panel of chemokines (18/21, 86%) and pro- and anti-inflammatory cytokines (11/19, 58%) in blood with a resulting signaling cascade leading to engagement of macrophages, dendritic cells, monocytes, NK cells and T cells without engagement of neutrophils. Although neutrophils do not appear to be engaged, a considerable number of chemokines that recruit other granulocytes such as eosinophils and basophils were also increased at 72h post infection with *L. interrogans*. Overall, the data suggest that prevention of dissemination of *L. biflexa* is associated with an early engagement of the innate immune response characterized by upregulation of a few chemokines that results in an efficacious phagocytic response without an overwhelming increase of pro-inflammatory cytokines. However, when macrophages fail to clear a pathogenic serovar such as *L. interrogans*, the adaptive response (T cells) is engaged to help out, but the resulting chemo-cytokine storm mediates a robust but non-resolving inflammatory response to pathogenic Leptospira that results in dissemination, kidney colonization, pathology and disease.

## Introduction

Leptospirosis is a neglected tropical disease with an incidence of a million annual human cases in temperate countries and more than 10 million during epidemic outbreaks in tropical areas (Costa et al., 2015; Roqueplo et al., 2019). About 10-15% patients develop severe leptospirosis with multi organ failure and high mortality rate (Cagliero et al., 2018). However, the exact global impact is unknown due to inadequate surveillance systems in most low-income countries (Guerra, 2013) and to under-reporting in developed countries (Fontes et al., 2015). Furthermore, the ubiquitous presence of pathogenic Leptospira in the environment and in different host species increases the zoonotic transmission of Leptospira (Cordonin et al., 2020). Thus, the disease has a severe socioeconomic impact due to infection of animals of agricultural interest (Schafbauer et al., 2019).

Leptospira pathogenesis depends on the virulence of the Leptospira serovar and how the immune system engages with the spirochete in the host (Cagliero et al., 2018). Despite extensive research to understand the nuances of host-pathogen interactions, several questions remain unanswered regarding the host recognition process. Prior reports have dissected differences in induction of proinflammatory cytokines and chemokines after infection of hamster and guinea pigs (Vernel-Pauillac and Merien, 2006; Lourdault et al., 2009; Fujita et al., 2015). However, studies on how the cellular arm of the immune system distinguishes between pathogenic and non-pathogenic Leptospira during early infection are very limited in mouse models (da Silva et al., 2009; Domingos et al., 2017). The first line of defense to pathogenic versus non-pathogenic Leptospira should be characterized by production of different signatures of attractant chemokines and cytokines that recruit specialized immune cells and drive inflammation (Santecchia et al., 2020) which ultimately leads to early elimination of the pathogen from the host and further modulate pathogenesis and disease progression.

Mice are reservoir hosts of pathogenic Leptospira. As such, this species is generally associated with resistance to infection (Adler and Faine, 1977). A possible factor underlying susceptibility to infection in humans may be a lack of recognition of leptospiral-LPS by human TLR4, whereas resistance in mice has been linked to production of antibody within 48-72h post infection (Adler and Faine, 1976) and the murine TLR4 ability to engage leptospiral-LPS (Werts et al., 2001; Nahori et al., 2005; Santecchia et al., 2020). However, mouse strains that express an impaired TLR4 in their immune cells (C3H-HeJ) are susceptible to lethal and sublethal leptospirosis (Pereira et al., 1998; Nally et al., 2005; Viriyakosol et al., 2006; Richer et al., 2015) and those that have been engineered to not express TLR4 (C57BL/6J-TLR4_ko_) succumb to infection (Chassin et al., 2009). We found recently that rather than leptospiral-LPS sensing, the presence of a fully functional TLR4 receptor in mice is necessary to control acute leptospirosis (Nair et al., 2021). In this study, we used the C3H-HeJ mouse model, which recapitulates susceptibility to leptospirosis, to analyze differences in cell-mediated immune markers engaged by pathogenic disseminators as well as those engaged by saprophytic non-disseminators during the earliest phase of infection with Leptospira, at 24h and 72h.

## Materials and Methods

### Animals

Male, 10-weeks old, C3H-HeJ mice were purchased from The Jackson Laboratory (Bar Harbor, ME). Male mice were chosen as it is reasonable to expect they should be more susceptible to Leptospira infection as we observed previously for male hamsters (Gomes et al., 2018). Animals were maintained and used in a pathogen-free environment in compliance with the University of Tennessee Health Science Center Institutional Animal Care and Use Committee Protocol no. 19-0062.

### Bacterial Strains and Infection

*Leptospira interrogans* serovar Copenhageni strain Fiocruz L1-130 (LIC), culture passage 2 after hamster infection, and cultured *Leptospira biflexa* serovar Patoc were used to infect mice. Pathogenic *L. interrogans* was originally isolated from a patient in Brazil. Non-pathogenic *L. biflexa* serovar Patoc was obtained from ATCC. Leptospira was cultured as previously described (Sullivan et al., 2017) and enumerated by dark-field microscopy (Zeiss USA, Hawthorne, NY) using a Petroff- Hausser counting chamber. About 10^8^ spirochetes were used to do intraperitoneal infections.

### q-PCR and RT-PCR

Quantification of Leptospira was performed using TAMRA probe and primers (Eurofins) to leptospiral 16s rRNA (*L. interrogans*) and 23s rRNA (*L. biflexa*) by qPCR. Primers and probes are described in **Table S1**. Isolation of DNA from blood was carried out using a NucleoSpin tissue kit (Clontech, Mountain View, CA) according to the manufacturer’s instructions and qPCR was run against a standard curve of 10^6^ to 1 *L. interrogans.* Extraction of total cellular mRNA from whole blood was done using Nucleospin RNA blood kit (Macherey Nagel) respectively. RNA purity was measured at wavelength A260/280 ratio using a Nanodrop instrument (Thermo Scientific). A high-capacity cDNA reverse transcription kit (Applied Biosystems) was used for cDNA preparation. TAMRA specific probes and primers for a panel of chemokines and cytokines as well as β-actin (Eurofins Genomics) were used (**Table S1**). Data were analyzed using the comparative CT method. Ct values over 38 were considered undetermined.

### Flow Cytometry

Single cell suspension from spleen were prepared after RBC lysis using a previously described protocol with minor modifications (Richer et al., 2015). Dead cells were eliminated using Live/dead cell stain during cell counting using a Luna cell counter (Logos Biosystems, South Korea). Approximately 10^6^ cells were seeded per well in a 96 well microtiter plate and blocked with anti-mouse CD16/32 antibody (1:50) for 15-20 min on ice in staining buffer. Surface staining was performed using appropriate primary conjugated antibody against different cell surface markers and incubated in the dark for 30 min at 4°C. Cells were washed twice with phosphate buffered saline (PBS, 1X; pH 7.4) and fixed with 4% Paraformaldehyde for 10 mins followed by a single PBS wash. Cells were resuspended in staining buffer, acquired on BioRad ZE5 Cell analyzer and data were analyzed using FlowJo software. The panel of fluorochrome conjugated antibodies (**Table S2**) and gating strategy (**Figure S1**) are described in **Supplementary Material**.

### Proteome Profile Array

Proteome Profiler Mouse Cytokine Array kit (Panel A, R&D Systems Inc., Minneapolis, MN, USA) was used for the detection of cytokines and chemokines (**Table S3**) in serum (Chondrou et al., 2020), following the manufacturer’s instructions. Nitrocellulose membrane contains spotted capture antibodies in duplicate. In brief, serum samples were mixed with a cocktail of 40 different biotinylated detection antibodies and then incubated with the Mouse Cytokine Array membrane. A complex is formed between the above mixture and the immobilized capture antibody in the membrane. A washing step is performed to remove unbound conjugate. Lastly, chemiluminescent and Streptavidin-HRP were used for detection of these conjugates, which is proportional to the bound cytokine quantity. Images were acquired using a Chemi-Doc image analyzer; the mean pixel density was measured using imageJ software and plotted using GraphPad Prism software. The mean pixel density values were normalized by subtracting the background of 3 membranes for each exposure (total of 3 exposures). Specifics on the mouse proteome profile array are presented in **Supplementary Material**, **Figures S2** and **S3**.

### Statistical Analysis

Statistical analysis was performed using unpaired t-test with Welch’s correction to analyze differences between non-infected and infected groups, and between infected *L. interrogans* and *L. biflexa* groups, α = 0.05.

## Results

### Infection With *L. interrogans* Leads to Spirochetemia in Contrast to Infection With *L. biflexa*

**Figure 1:**
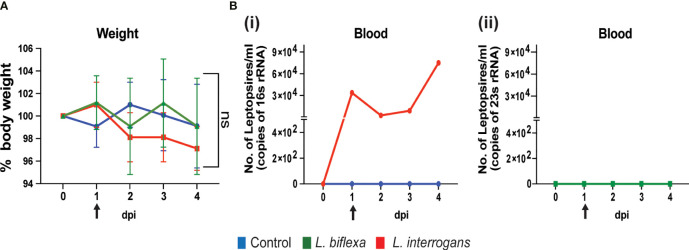
Body weight and dissemination of Leptospira in blood of infected mice. Male C3H-HeJ mice were infected with 10^8^ *L. interrogans* serovar Copenhageni strain Fiocruz L1-130 and *L. biflexa* serovar Patoc whereas control mice received PBS. Panel **(A)** shows 4-day body weight measurements of mice injected with *L. interrogans* (red), *L. biflexa* (green), and PBS control (blue). Panel **(B)** depicts the total Leptospira burden in blood of *L. interrogans* (i) and *L. biflexa* (ii) infected mice over 4 days post infection by qPCR. n=4 mice per group. Arrow indicates the day of infection. Statistical analysis was performed by unpaired t-test with Welch’s correction between control and infected groups. ns, non-significance.

We inoculated mice with 10^8^ spirochetes and measured weight-loss as well as Leptospira load in blood by qPCR for 4 days. As expected, within the first 4 days of infection there is no significant change in body weight between the groups (**Figure 1A**). In addition, dissemination of pathogenic *L. interrogans* in blood was detected from day 1 to day 4 post infection (**Figure 1B** i) whereas no dissemination occurred for non-pathogenic *L. biflexa* (**Figure 1B** ii) infected mice.

### *L. interrogans* Infection Led to Increased Circulation of Chemokines and Cytokines in Serum Mostly at 72h, Whereas *L. biflexa* Infection Led to Increased Circulation of a Few Chemokines Mostly at 24h

**Figure 2:**
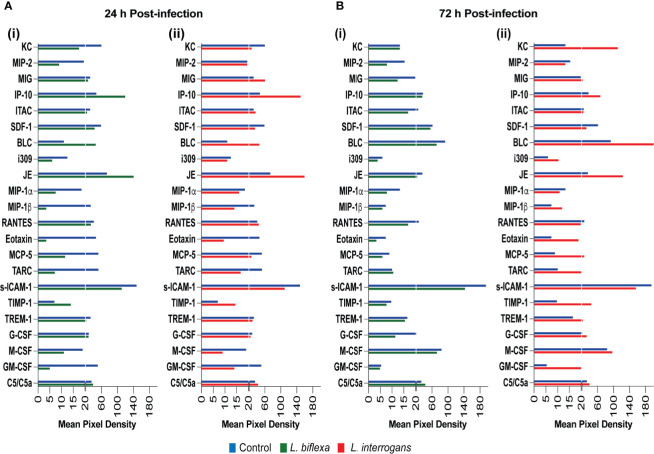
Proteome profiling of chemokines circulating in serum from mice infected with *L. interrogans, L. biflexa* and PBS controls. Detection of chemokine proteins in pooled serum (n=4 mice), represented as mean pixel density at 24h **(A)** and 72h **(B)** post infection. Sub-panel (i) depicts representative graphs of *L. biflexa* vs control, whereas (ii) depicts *L. interrogans* vs control. The chemiluminescence was measured and the mean pixel density value was determined using ImageJ software. The values were normalized by subtracting the background of 3 membranes for each exposure for a total of 3 exposures. Data represents the average pixel density between the two spots for each immune marker after subtraction of the normalized background.

**Figure 3:**
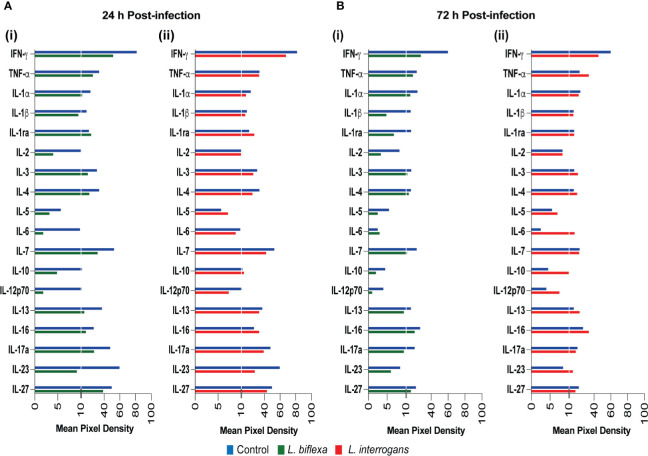
Proteome profiling of cytokines circulating in serum from mice infected with *L. interrogans, L. biflexa* and PBS controls. Detection of cytokine proteins in pooled serum (n=4 mice), represented as mean pixel density at 24h **(A)** and 72h **(B)** post infection. Sub-panel (i) depicts representative graphs of *L. biflexa* vs control, whereas (ii) depicts *L. interrogans* vs control. The chemiluminescence was measured and the mean pixel density value was determined using ImageJ software. The values were normalized by subtracting the background of 3 membranes for each exposure for a total of 3 exposures. Data represents the average pixel density between the two spots for each immune marker after subtraction of the normalized background.

We quantified circulating immune mediators in serum using a mouse Proteome Profile Array (**Figures 2**, **3** and **Tables 1**, **2**, **4**; **Table S3**) targeting the following 40 proteins: KC/CXCL1, MIP-2/CXCL2, MIG/CXCL9, IP-10/CXCL10, I-TAC/CXCL11, SDF-1/CXCL12, BLC/CXCL13, I-309/CCL1, JE/MCP-1/CCL2, MIP-1α/CCL3, MIP-1β/CCL4, RANTES/CCL5, Eotaxin/CCL11, MCP-5/CCL12, TARC/CCL17, sICAM-1, TIMP-1, TREM-1, G-CSF, M-CSF, GM-CSF, C5/C5a, IFN-γ, TNF-α, IL-1α, IL-1β, IL-1ra, IL-2, IL-3, IL-4, IL-5, IL-6, IL-7, IL-10, IL-12p70, IL-13, IL-16, IL-17a, IL-23, IL-27. At 24h post infection, we found that *L. biflexa* increased secretion of 4/21 (19%) chemokines in serum as compared to control, whereas 17/21 (81%) were decreased; in addition, no cytokines were increased, whereas 16/18 (89%) were decreased. At 24h, *L. interrogans* infection increased secretion of 7/21 (33%) chemokines in serum, whereas 11/21 (52%) were decreased; furthermore, 2/18 (11%) cytokines were increased whereas 10/18 (56%) were decreased. Specifically, *L. interrogans* induced MIG, I-TAC, RANTES and the cytokines IL-1ra and IL-16 in addition to the 4 chemokines also induced by *L. biflexa*: IP-10, BLC, JE/MCP-1 and TIMP-1 (**Figure 2A**, **Figure 3A**; **Tables 1**, **2** and Summary in **Table 4**). At 72h post infection with *L. biflexa*, no chemo-cytokine activity was increased in serum as compared to control; furthermore, 12/21 (57%) chemokines as well as 12/18 (67%) cytokines were decreased. At 72h, *L. interrogans* infection increased 14/21 (67%) pro-inflammatory chemokines (KC, IP-10, BLC, I-309 JE/MCP-1, MIP-1β, Eotaxin, MCP-5, TARC, TIMP-1, TREM-1, G-CSF, M-CSF and GM-CSF) and 9/18 (50%) cytokines in serum as compared to control (TNF-α, IL-3, IL-4, IL-6, IL-10, IL-12p70, IL-13, IL-16 and IL-23); in addition, 3/21 (14%) chemokines (RANTES, SDF-1 and ICAM-1) and 2/18 (11%) cytokines (IFN-γ and IL-27) were decreased (**Figure 2B**, **Figure 3B**; **Tables 1**, **2** and Summary in **Table 4**). Both *L. interrogans* and *L. biflexa* induced increased concentration of complement factor C5/C5a in serum in comparison with the control.

**Table 1:** Chemokines and complement factor affected during early infection with pathogenic and non-pathogenic Leptospira in comparison with control.

Name	Genetic expression (Whole blood)				Protein expression (Serum)			
	*L. biflexa*		*L. interrogans*		*L. biflexa*		*L. interrogans*	
	24 h	72 h	24 h	72 h	24 h	72 h	24 h	72 h
**CHEMOKINES**								
KC/CXCL1	und	und	und	und	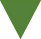	**≈**	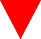	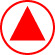
MIP-2/CXCL2	ns	ns	ns	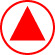	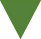	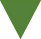	**≈**	**≈**
MIG/CXCL9	ns	ns	ns	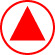	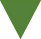	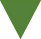	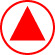	**≈**
IP-10/CXCL10	ns	und	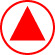	und	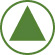	**≈**	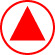	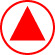
I-TAC/CXCL11					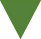	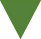	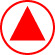	**≈**
SDF-1/CXCL12					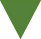	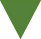	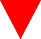	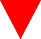
BLC/CXCL13	und	und	und	und	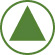	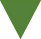	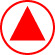	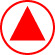
I-309/CCL1					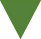	**≈**	**≈**	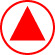
JE/MCP-1/CCL2	und	und	und	und	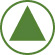	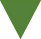	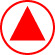	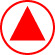
MIP-1α/CCL3					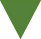	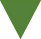	**≈**	**≈**
MIP-1β/CCL4					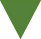	**≈**	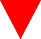	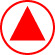
RANTES/CCL5	ns	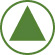	ns	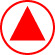	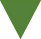	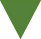	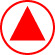	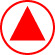
Eotaxin/CCL11					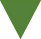	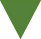	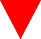	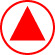
MCP-5/CCL12	und	und	und	und	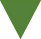	**≈**	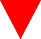	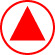
TARC/CCL17					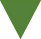	**≈**	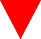	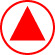
sICAM-1					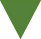	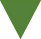	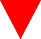	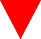
TIMP-1	und	und	und	und	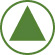	**≈**	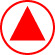	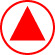
TREM-1					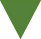	**≈**	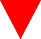	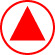
G-CSF	und	und	und	und	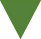	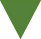	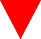	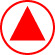
M-CSF					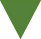	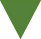	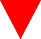	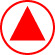
GM-CSF	und	und	und	und	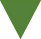	**≈**	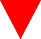	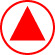
**COMPLEMENT FACTOR**								
Complement C5/C5a					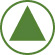	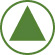	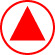	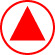

L. biflexa (green) and L. interrogans (red) compared to control at 24h and 72h post infection; **≈**, represents equivalent (mean pixel density ± 3); ns, represents non-significance and und, represents undetermined.

**Table 2:** Cytokines affected during early infection with pathogenic and non-pathogenic Leptospira in comparison with control.

Name	Genetic expression				Protein expression			
	(Whole blood)				(Serum)			
	*L. biflexa*		*L. interrogans*		*L. biflexa*		*L. interrogans*	
	24h	72h	24h	72h	24h	72h	24h	72h
**CYTOKINES**								
IFN-γ	und	und	und	und	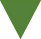	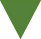	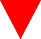	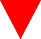
TNF-α	ns	ns	ns	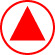	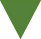	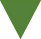	**≈**	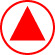
IL-1α					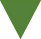	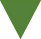	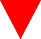	**≈**
IL-1β	ns	ns	ns	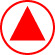	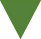	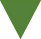	**≈**	**≈**
IL-1ra					**≈**	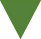	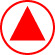	**≈**
IL-2	und	und	und	und	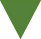	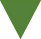	**≈**	**≈**
IL-3					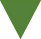	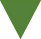	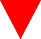	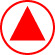
IL-4	und	und	und	und	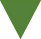	**≈**	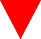	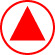
IL-5					**≈**	**≈**	**≈**	**≈**
IL-6	ns	ns	ns	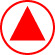	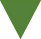	**≈**	**≈**	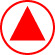
IL-7					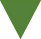	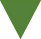	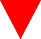	**≈**
IL-10	und	und	und	und	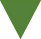	**≈**	**≈**	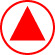
IL-12p70	und	und	und	und	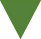	**≈**	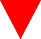	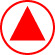
IL-13					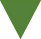	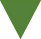	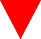	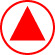
IL-16	ns	ns	ns	ns	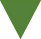	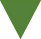	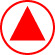	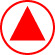
IL-17a	und	und	und	und	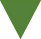	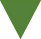	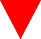	**≈**
IL-18	ns	ns	ns	ns				
IL-23	und	und	und	und	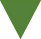	**≈**	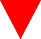	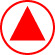
IL-27					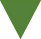	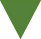	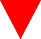	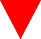

L. biflexa (green) and L. interrogans (red) compared to control at 24h and 72h post infection; **≈**, represents equivalent (mean pixel density ± 3); ns, represents non-significance and und, represents undetermined.

### *L. interrogans* Infection Increased Levels of Chemokine and Cytokine mRNA Mediators in Whole Blood at 72h Post Infection, Whereas *L. biflexa* Remained Mostly Unchanged

**Figure 4:**
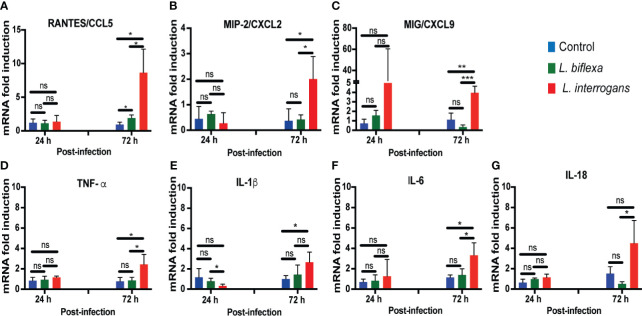
Genetic expression levels of chemo-cytokines in whole blood at 24h and 72h post infection with *L. biflexa* and *L. interrogans*. Infection was performed with pathogenic and non-pathogenic *Leptospira* in C3H-HeJ mice *via* intraperitoneal route with 10^8^ bacteria for 24h and 72h. PBS was administered in control mice. We measured genetic expression of inflammatory signatures by RT-PCR (Panels **A–G**) from whole blood. Statistical significance was determined by unpaired t-test with Welch’s correction between control and infected groups, where *p<0.05, **p<0.01, ***p<0.001, ns, non-significance; n= 3-4 mice per group. Data represents one of two independent experiments.

We quantified expression of mRNA of 23 transcripts KC/CXCL1, MIP-2/CXCL2, MIG/CXCL9, IP10/CXCL10, BLC/CXCL13, JE/MCP-1/CCL2, RANTES/CCL5, MCP-5/CCL12, TIMP-1, G-CSF, GM-CSF, IFN-γ, TNF-α, IL-1β, IL-2, IL-4, IL-6, IL-10, IL-12, IL-16, IL-17a, IL-18, IL-23 in cells from whole blood (**Figure 4**; **Tables 1**, **2**, **4** and **Table S3**). Only immune markers with detectable levels of mRNA at 24h and 72h post infection were plotted in **Figure 4**. Significant differences between genetic expression of immune mediators were detected at 72h but not at 24h post infection with *L. interrogans*, except for an increase of IP-10 between *L. interrogans* and control (**Table 1**) at 24h, and a decrease in IL-1β between *L. biflexa* and *L. interrogans* infected mice (**Figure 4E**) at 24h. At 72h post-infection, RANTES was the only chemokine increased between *L. biflexa* and control groups (**Figure 4A** and **Table 1**). Between *L. interrogans* and control groups, the chemokines RANTES, MIP-2, and MIG, and the cytokines TNF-α, IL-1β, and IL-6 were increased (**Figures 4A–F** and **Tables 1** and **2**). IL-18 was increased in *L. interrogans* compared to *L. biflexa* but it was not different from the control (**Figure 4G**). The other 15 immune markers tested in mRNA purified from whole blood (KC, BLC, JE/MCP-1, MCP-5, TIMP-1, G-CSF, GM-CSF, IFN-γ, IL-2, IL-4, IL-10, IL-12, IL-16, IL-17a, IL- 23) were either not significant or not detected.

### *L. interrogans* Infection Led to Engagement of All Myeloid Cell Populations in Spleen Except Neutrophils, Whereas *L. biflexa* Infection Led to Engagement of Resident Macrophages, Dendritic Cells, NK Cells and Neutrophils

**Figure 5:**
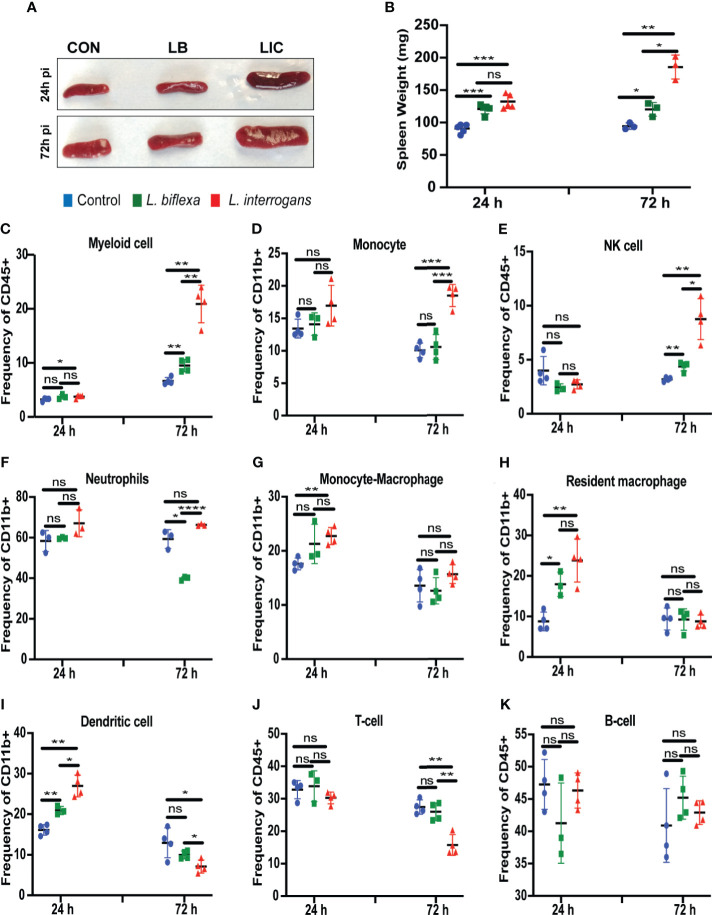
Spleen morphometry and flow cytometric analysis of immune cells after infection. **(A)** depicts clear *L. interrogans* induced splenomegaly, while *L. biflexa* spleen appears to retain normal splenic architecture as do controls at 24h and 72h pi. **(B)** is the representation of respective splenic weight in milligrams, differences in weight were measured both for *L. biflexa* and *L. interrogans*. Statistical analysis between groups was done by unpaired t-test with Welch’s correction, *p<0.05, **p<0.01, ***p<0.001, n= 3-5 mice per group. **(C–K)** represent different immune cell population in spleen at 24h and 72h pi. Myeloid cells like monocytes **(D)**, neutrophils **(F)**, monocyte-macrophage **(G)**, resident macrophage **(H)**, and dendritic cells **(I)** were determined based on CD11b+ frequency. Myeloid cells **(C)**, NK-cell **(E)**, T-cell **(J)** and B-cell **(K)** were measured in terms of CD45+ frequency. Statistical analysis was done by unpaired t-test with Welch’s correction between control and infected groups, where *p<0.05, **p<0.01, ***p<0.001, ****p<0.0001, ns, non-significance; n=4 mice per group. Data represents one of three independent experiments.

Mice infected with *L. interrogans* had enlarged spleens both at 24h and 72h post infection as compared to controls, whereas differences in spleens harvested from *L. biflexa* infected mice were less apparently enlarged as observed visually (**Figure 5A**), and by weight measurement (**Figure 5B**). We phenotyped the immune cells contributing with the elevated expression of inflammatory signatures during the earliest phase of infection by flow cytometry (**Figures 5C–K** and **Table 3**). We found that at 24h post infection, myeloid cells (**Figure 5C**), monocyte-macrophages (**Figure 5G**), resident macrophages (**Figure 5H**) and dendritic cells (**Figure 5I**) were significantly increased in *L. interrogans* infected groups compared to controls; at 72h post infection, myeloid cells (**Figure 5C**), monocytes (**Figure 5D**), NK cells (**Figure 5E**) were significantly increased and, dendritic cells (**Figure 5I**) and T cells (**Figure 5J**) were significantly decreased whereas neutrophils (**Figure 5F**) and macrophages (**Figure 5G, H**) were not engaged in *L. interrogans* infected groups compared to controls. Between *L. biflexa* and control groups, at 24h post-infection, resident-macrophages (**Figure 5H**) and dendritic cells (**Figure 5I**) were increased, and at 72h post infection, myeloid cells (**Figure 5C**) and NK cells (**Figure 5E**) where increased, whereas neutrophils (**Figure 5F**) were decreased. A comparison between *L. interrogans* and *L. biflexa* at 72h post infection shows that myeloid cells (**Figure 5C**), monocytes (**Figure 5D**), NK cells (**Figure 5E**) and neutrophils (**Figure 5F**) were significantly increased in *L. interrogans*, whereas dendritic cells (**Figure 5I**) and T cells (**Figure 5J**) are significantly decreased. No differences in B cells were observed between *L. interrogans, L. biflex*a and controls at any time points (**Figure 5K**).

**Table 3:** Immune cells engaged during infection with pathogenic and non-pathogenic Leptospira at 24h and 72h post-infection in comparison with control.

Immune Cell Phenotypes (Spleen)				
Name	*L. biflexa*		*L. interrogans*	
	24 h	72 h	24 h	72 h
Myeloid cell	ns	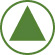	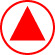	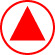
Monocyte	ns	ns	ns	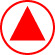
Monocyte-Macrophage	ns	ns	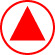	ns
Resident Macrophage	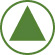	ns	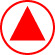	ns
Neutrophil	ns	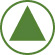	ns	ns
Dendritic cell	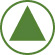	ns	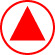	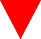
Natural Killer cells	ns	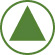	ns	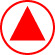
B-cell	ns	ns	ns	ns
T-cell	ns	ns	ns	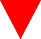

L. biflexa (green) and L. interrogans (red) compared to control at 24h and 72h post-infection; ns, indicates non-significance.

A summary of all the factors measured such as protein circulating in serum, cellular mRNA from whole blood and immune cells homing to the secondary lymphoid organ - spleen - that orchestrate the inflammatory immune response to pathogenic *L. interrogans* and saprophytic *L. biflexa* is presented in **Table 1** (21 chemokines and Complement component 5 - C5/C5a), **Table 2** (19 cytokines), **Table 3** (9 immune cell types), **Table 4** (overall summary) and **Table S3** (immune markers and cell function). Over the two time points, *L. biflexa* infection increased secretion of Complement component 5 (C5/C5a), 5/21 (24%) chemokines and 0/19 (0%) cytokines in serum and blood cells, and engaged 5/9 (56%) immune cell types in spleen. In contrast, *L. interrogans* increased secretion of C5/C5a, 18/21 (86%) chemokines and 11/19 (58%) inflammatory cytokines in serum and blood cells, and engaged 6/9 (67%) immune cell types in spleen.

**Table 4:** Summary of immune markers and cells involved in responses to *L. biflexa* and *L. interrogans* infection.

CHEMOKINES											
	Genetic Expression in Whole Blood Nr (%) [n=11]				Protein Expression in Serum Nr (%) [n=21]					Genetic + Protein Expression Nr (%) [n=21]	
	*L. biflexa*		*L. interrogans*		*L. biflexa*		*L. interrogans*			*L. biflexa*	*L. interrogans*
	24h	72h	24h	72h	24h	72h	24h	72h		Total	Total
Increased (BL+S)	–	1(9%)	1(9%)	3(27%)	4(19%)	–	7(33%)	14(67%)		**5(24%)**	**18(86%)**
Decreased (BL+S)	–	–	–	–	17(81%)	12(57%)	11(52%)	3(14%)		**19(91%)**	**12(57%)**
Equivalent (S)	–	–	–	–	–	9(43%)	3(14%)	4(19%)		9(43%)	5(24%)
Undetermined (BL)	7(64%)	8(73%)	7(64%)	8(73%)	–	–	–	–		8(38%)	8(38%)
Non-significant (BL)	4(36%)	2(18%)	3(27%)	–	–	–	–	–		4(19%)	3(14%)
**CYTOKINES**											
	**Genetic Expression in Whole Blood Nr (%) [n=12]**				**Protein Expression in Serum Nr (%) [n=18]**					**Genetic + Protein Expression Nr (%) [n=19]**	
	***L. biflexa***		***L. interrogans***		***L. biflexa***		***L. interrogans***			***L. biflexa***	***L. interrogans***
	**24h**	**72h**	**24h**	**72h**	**24h**	**72h**	**24h**	**72h**		**Total**	**Total**
Increased (BL+S)	–	–	–	3(25%)	–	–	2(11%)	9(50%)		**0 (0%)**	**11(58%)**
Decreased (BL+S)	–	–	–	–	16(89%)	12(67%)	10(56%)	2(11%)		**17(90%)**	**10(53%)**
Equivalent (S)	–	–	–	–	2(11%)	6(33%)	6(33%)	7(39%)		7(37%)	10(53%)
Undetermined (BL)	7(58%)	7(58%)	7(58%)	7(58%)	–	–	–	–		7(37%)	7(37%)
Non-significant (BL)	5(42%)	5(42%)	5(42%)	2(17%)	–	–	–	–		5(26%)	5(26%)
**IMMUNE CELL PHENOTYPES in SPLEEN** **Nr (%) [n=9]**											
	***L. biflexa***						***L. interrogans***				
	**24h**		**72h**		**Total**		**24h**		**72h**		**Total**
Increased	2(22%)		3(33%)		**5(56%)**		4(44%)		3(33%)		**6(67%)**
Decreased	–		–		**0(0%)**		–		2(22%)		**2(22%)**
Non-significant	7(78%)		6(67%)		9(100%)		5(56%)		4(44%)		7(86%)

Legend: BL, Blood; S, Serum; Bold, Total up- or down-regulated at 24h and 72h with common results at both timepoints being counted once.

## Discussion

The engagement of the host immune system with pathogenic and non-pathogenic serovars of Leptospira at the point of entry defines the outcome of infection. In this study, we quantified immune cell mediators circulating in serum and genetic expression of chemo/cytokines in whole blood of C3H-HeJ mice infected with pathogenic and saprophytic Leptospira (*L. interrogans* and *L. biflexa*) and correlated these circulating signatures with the immune cell populations enriched in the secondary lymphoid organ (spleen) during the earliest phase of infection. We analyzed two timepoints (24h and 72h) within the first week of infection when *Leptospira interrogans* is known to circulate in blood and there are no weight differences - a clinical score of disease progression - in infected C3H-HeJ mice [**Figure 1** (Richer et al., 2015; Nair et al., 2020)].

We found that infection with *L. biflexa* was generally characterized by induction of chemoattractant chemokines (IP-10, JE/MCP-1) that engage resident macrophages and dendritic cells in spleen at 24h post infection which was replaced by an increase of Natural Killer (NK) cells and a decrease of neutrophils at 72h post infection (**Figures 2** and **5**). Although RANTES and BLC chemoattractants were increased there was no evidence of engagement of T cells and B cells, respectively. The marked decrease in neutrophils frequency in spleen from mice infected with *L. biflexa* could be explained by its rapid recruitment to the spirochete point of entry as it is known that neutrophils phagocytize and destroy saprophytic Leptospira (Isogai et al., 1989; Scharrig et al., 2015; Charo et al., 2019). An efficacious phagocytic response engaged by resident macrophages (Toma et al., 2011), and neutrophils (Scharrig et al., 2015) without an overwhelming increase of pro-inflammatory cytokines contributes to elimination of the spirochete at the point of entry preventing Leptospira dissemination and absence of pathology and disease. However, it is not clear which immune mediators recruited neutrophils given that the neutrophil chemoattractants tested (KC, MIP-2 and IL-1α) were downregulated at 24h and 72h post infection with *L. biflexa*. Regarding the other two cell types (NK cells and dendritic cells) involved in clearance of *L. biflexa*, both are induced by JE/MCP-1 which was increased in serum at 24h post-infection. The protective effects of NK cells from vaccinated bovines (Zuerner et al., 2011) and from serum-thymic factor treated gerbils (Yukawa et al., 1994) have been described. Thus, we speculate that NK cells may directly eliminate cells that have phagocytized *L. biflexa*. Both pathogenic and non-pathogenic Leptospira induced maturation of human dendritic cells and production of pro-inflammatory cytokines (Gaudart et al., 2008; Santecchia et al., 2020). In another study, liposomes prepared from *L. biflexa* lipids activated bone marrow derived dendritic cells that released pro-inflammatory cytokines and induced strong humoral and cytotoxic T cell responses (Faisal et al., 2011). In our study, we did not detect any pro-inflammatory cytokines in serum or blood cells of C3H-HeJ mice infected with *L. biflexa*. Thus, the role played by these professional antigen presenting cells is unclear, but it may be related to the recruitment of cytotoxic T cells.

In contrast to *L. biflexa*, infection with *L. interrogans* was characterized by a substantial induction of attractant chemokines whose function is to recruit myeloid phagocytic cells and pro- and anti-inflammatory cytokines whose main function is to engage lymphoid cells (**Figures 2** **–** **5**). The frequency of macrophages and dendritic cells was likely driven by MIG, IP-10 and JE/MCP-1 chemokines increased in blood at 24h post-infection. These cells were replaced by a subsequent increase in monocytes and Natural Killer cells and a decrease of dendritic cells driven by IP-10, I-309, JE/MCP-1, MIP-1β and MCP-5 at 72h post-infection. T cell recruitment may have been driven by IP-10, I-309, RANTES, MCP-5 and TARC at 72h post infection. As observed for *L. biflexa*, BLC which is involved in B cell recruitment was increased in serum of *L. interrogans* infected mice but differences in B cell frequency in spleen were not significant at this timepoint. This suggests engagement of the adaptive arm of the immune system as early as 24h-72h post infection with *L. interrogans* as described by others (Adler and Faine, 1976). Chemokine proteome arrays in *L. interrogans* infected mice have been done by other groups (Domingos et al., 2017; Silva et al., 2020). Differences observed in the panel of chemokines detected can be explained by differences in methodology in that we quantified circulating chemokines (serum and blood cells) and they quantified chemokines in spleen/lung (Domingos et al., 2017) and in liver/kidney (Silva et al., 2020). Nevertheless, MIG/CXCL9, IP-10/CXCL10, I-TAC/CXCL11, BLC/CXCL13 and JE/MCP-5/CCL2 detected in kidney of C3H-HeJ mice at 24h post infection (Silva et al., 2020) was also detected in serum/blood cells in our study.

Neutrophil chemoattractants, KC and MIP-2, were increased in the serum proteome and in whole blood gene transcripts of *L. interrogans* infected mice. However, neutrophils were not increased or decreased in spleen. Earlier studies have shown that neutrophils do not phagocytize pathogenic *Leptospira* (Isogai et al., 1989; Scharrig et al., 2015; Charo et al., 2019; Santecchia et al., 2020). Others have shown that macrophages, but not neutrophils, infiltrate organs of C3H-HeJ mice infected with *L. interrogans* (Chen et al., 2017). This may explain the lack of engagement of neutrophils in *L. interrogans* infected C3H-HeJ mice in our study.

Discrepant results between serum protein and genetic expression in blood may reflect the limited number of cell types circulating in blood at the time of blood collection, whereas serum receives proteins from all immune cell types from different organs and tissues. This may explain why we detected a more comprehensive panel of immune markers in serum than in transcripts from whole blood. Nevertheless, it is interesting to note that the immune markers detected by genetic expression represent the overall engagement of all arms of the immune cell response: MIP-2 (neutrophils), MIG and TNF-α (macrophages/NK cells), IP-10 (monocytes, macrophages, dendritic cells, NK cells and T cells), RANTES (T cells, eosinophils, basophils), IL-1β (immune cell proliferation, differentiation and apoptosis) and IL-6 (pro-inflammatory and regulatory-T cell suppression).

As expected, and in contrast to *L. biflexa* infection, *L. interrogans* induced a strong adaptive immune response with production of proinflammatory cytokines TNF-α (innate immune alarm), IL-1ra (inhibitor of IL-1), IL-3, IL-4 (Th2), IL-6 (pro- and anti-inflammatory, Treg suppression), IL-10 (anti-inflammatory), IL-12p70 (NK and Th1 cell function), IL-13 (Th2, IgE antibody), IL-16 (activated T cells, dendritic cells and eosinophils), and IL-23 (Th17 expansion, induces IL-6). Furthermore, a considerable number of chemokines that recruit eosinophils and basophils (RANTES, Eotaxin, MCP-5, G-CSF, GM-CSF) were also increased at 72h post infection with *L. interrogans*. Although neutrophils do not appear to be engaged by *L. interrogans*, these other granulocytes may contribute to inflammation.

## Conclusion

The stark differences in expression of chemoattractant chemokines and pro- and anti-inflammatory cytokines can be correlated with pathogenesis. These data suggest that prevention of dissemination of *L. biflexa* is associated with an early engagement of the innate immune system characterized by secretion of a few chemoattractants that results in an efficacious phagocytic response by resident macrophages and neutrophils, which are helped by dendritic and Natural Killer cells without an overwhelming increase of pro-inflammatory cytokines. However, when macrophages fail to clear a pathogenic serovar such as *L. interrogans*, monocytes, NK cells, T cells and possibly other granulocytes (eosinophils and basophils) may be recruited to help out. The resulting chemo-cytokine storm mediates a robust but non-protective inflammatory response to pathogenic Leptospira (Cagliero et al., 2018) which results in dissemination, kidney colonization, pathology and disease.

## Data Availability Statement

The original contributions presented in the study are included in the article/**Supplementary Material**. Further inquiries can be directed to the corresponding author.

## Ethics Statement

The animal study was reviewed and approved by University of Tennessee Health Science Center Institutional Animal Care and Use Committee.

## Author Contributions

AS and SK contributed equally. MGS is the corresponding author who conceptualized the project, procured funding, designed experiments, data analysis, prepared figures and wrote the manuscript. AS and SK designed and performed the experiments, assisted in writing the manuscript, data analysis and figure preparation. All authors contributed to the article and approved the submitted version.

## Funding

This work was supported by Public Health Service grants R43AI136551, R21AI142129 and R43AI155211 to MGS from the National Institutes of Health, National Institute of Allergy and Infectious Diseases.

## Conflict of Interest

The authors declare that the research was conducted in the absence of any commercial or financial relationships that could be construed as a potential conflict of interest.
