# Supplementary material for: Inflammatory Signatures of Pathogenic and Non-Pathogenic Leptospira Infection in Susceptible C3H-HeJ Mice
Source: Front Cell Infect Microbiol. 2021 Jun 24;11:677999. doi: 10.3389/fcimb.2021.677999 (PMC8264587; doi:10.3389/fcimb.2021.677999)
Supplement: Supplementary file 1 [file DataSheet_1.docx]

**Table S1. List of primers and probes used in q-PCR and RT-PCR.**

| **Gene** | **Forward Primer** | **Reverse Primer** | **TAMRA Probe** |
| --- | --- | --- | --- |
| KC/CXCL1 | CGAGGCTTGCCTTGACCCTGAA | GGGACACCTTTTAGCATCTT | CCCTTGGTTCAGAAAATTGTCCA |
| MIP-2/CXCL2 | TGACTTCAAGAACATCCAGAGCTT | CTTGAGAGTGGCTATGACTTCTGTC | TGACGCCCCCAGGACCCCA |
| MIG/CXCL9 | GAACCCTAGTGATAAGGAATGCA | CTGTTTGAGGTCTTTGAGGGATT | CATCAGCACCAGCCGAGGCACG |
| IP-10/CXCL10 | AGTGCTGCCGTCATTTTCTG | ATTCTCACTGGCCCGTCAT | AGTCCCACTCAGACCCAGCAGG |
| BLC/CXCL13 | TGGCCAGCTGCCTCTCTC | TTGAAATCACTCCAGAACACCTACA | AGGCCACGGTATTCTGGAAGCCCAT |
| JE/MCP-1/CCL2 | CTTCTGGGCCTGCTGTTCA | CCAGCCTACTCATTGGGATCA | CTCAGCCAGATGCAGTTAACGCCCC |
| RANTES/CCL5 | AGTGCTCCAATCTTGCAGTCGT | CTTCTTCTCTGGGTTGGCACACACT | TTGTCACTCGAAGGAACCG |
| MCP-5/CCL12 | ACATGAAGATTTCCACACTTCTATGC | CAGCCAATACCTGAGGACTGATG | CCTGCTCATAGCTACCA |
| TIMP-1 | CAGAACCGCAGTGAAGAGTTTC | GCTGCAGGCACTGATGTG | ATCACGGGCCGCCTAA |
| G-CSF | GCTGCTGCTGTGGCAAAGT | AGCCTGACAGTGACCAGG | CACTATGGTCAGGACGAGAGGCCGTT |
| GM-CSF | GCCATCAAAGAAGCCCTGAA | GCGGGTCTGCACACATGTTA | ACATGCCTGTCACATTGAATGAAGAGGTAGAAG |
| IFN-γ | CAAGTGGCATAGATGTGGAAGAAA | CTGGCTCTGCAGGATTTTCA | GGAGGAACTGGCAAAAFFATGGTGAC |
| TNF-α | CACACTCAGATCATCTTCTCAAAAT | AAGGTACAACCCATCGGCTGGCA | AGCCTGTAGCCCACGTCGTAGCAAAC |
| IL-1β | AACCAACAAGTGATATTCTCCATG | GATCCACACTCTCCAGCTGC | TGTGTAATGAAAGACGGCACACCCACC |
| IL-2 | GAATGGAATTAATAATTACAAGAATC | ATGTTGTTTCAGATCCCTTTAGTTCCAGA | ACATGCCCAAGAAGGCCACAGAACTG |
| IL-4 | TGTACCAGGAGCCATATCCA | TTCTTCGTTGCTGTGAGGAC | ATCCATCTCCGTGCATGGCG |
| IL-6 | TCCTACCCCAATTTCCAATGC | TGAATTGGATGGTCTTGGTCC | CAGATAAGCTGGAGTCACAGAAGGAGTGG |
| IL-10 | CCAGCTGGACAACATACTGC | TGGATCATTTCCGATAAGGC | TGGCAACCCAAGTAACCCTTAAAGTCC |
| IL-12 | GGAAGCACGGCAGCAGAATA | AACTTGAGGGAGAAGTAGGAATGG | CATCATCAAACCAGACCCGCCCAA |
| IL-16 | ACAGAAGGAGAGTCAAGGAGGA | GCAGTCGGAAATTCTAACCCAAG | CCAGCCCAGAGACTCCAGCATCCC |
| IL-17a | ACCGCAATGAAGACCCTGAT | TCCCTCCGCATTGACACA | CTGGGAAGCTCAGTGCCGCCAC |
| IL-18 | CAGGCCTGACATCTTCTGCAA | TCTGACATGGCAGCCATTGT | CTCCAGCATCAGGACAAAGAAAGCCG |
| IL-23 | TGTGCCCCGTATCCAGTGT | CGGATCCTTTGCAAGCAGAA | TGTGACCCACAAGGACTCAAGGACAACA |
| β-actin | CCACAGCTGAGAGGGAAATC | CCAATAGTGATGACCTGGCCG | GGAGATGGCCACTGCCGCATC |
| Leptospiral 16s rRNA | CCCGCGTCCGATTAG | TCCATTGTGGCCGAACAC | CTCACCAAGGCGACGATCGGTAGC |
| Leptospiral 23s rRNA | ACAATCTTACCAAACCCTATC | TTACCACTTAGCGTAGATTT | TCCGAATACTGTAACTTGAAGTACTGCA |

**Table S2. List of antibodies used in flow cytometry**

| **Marker** | **Fluorophore** | **Company** |
| --- | --- | --- |
| CD45 | Brilliant Violet 605 | Biolegend |
| CD3 | Violet Fluor 450 | TONBO biosciences |
| CD19 | Alexa Fluor 700 | Biolegend |
| CD49b | PE Dazzle 594 | Biolegend |
| F4/80 | Brilliant Violet 785 | Biolegend |
| CD11b | APC | TONBO biosciences |
| CD11c | APC-Cy7 | TONBO biosciences |
| Ly6C | FITC | Biolegend |
| Ly6G | PE | Biolegend |
| MHC-II | PE-Cy7 | Biolegend |

**Table S3. List of immune markers and cells involved in innate and/or adaptive immune response and their respective functions**

| **Name** | **Function and role in immunity** | **Type of Immunity** | **Tested for genetic expression** | **Tested for protein expression** |
| --- | --- | --- | --- | --- |
| **CHEMOKINES** | | | | |
| KC/CXCL1 | Keratinocytes-derived Chemokine is a major chemoattractant of neutrophils. | Innate | ✓ | ✓ |
| MIP-2/CXCL2 | Macrophage Inflammatory Protein 2 is a major chemoattractant of neutrophils. | Innate | ✓ | ✓ |
| MIG/CXCL9 | Monokine Induced by Gamma interferon recruits immune cells, such as cytotoxic lymphocytes (CTLs), natural killer (NK) cells, NKT cells, and macrophages. | Innate and adaptive | ✓ | ✓ |
| IP-10/CXCL10 | Interferon gamma-induced Protein 10 is responsible for chemoattraction of monocytes/macrophages, T cells, NK cells and dendritic cells. | Innate and adaptive | ✓ | ✓ |
| I-TAC/CXCL11 | Interferon-inducible T-cell Alpha Chemoattractant is involved in activated T-cell chemotaxis and recruitment of NK cells. | Adaptive |  | ✓ |
| SDF-1/CXCL12 | Stromal cell-Derived Factor 1 acts as a lymphocyte chemoattractant and involved in hematopoietic stem cells homing. | Innate and adaptive |  | ✓ |
| BLC/CXCL13 | B Lymphocyte Chemoattractant is involved in B cell recruitment. | Adaptive | ✓ | ✓ |
| I-309/CCL1 | I-309 is involved in recruitment of monocytes, macrophages, T regulatory, Th2 cells and NK cells to the site of action. | Innate and adaptive |  | ✓ |
| JE/MCP-1/CCL2 | Monocyte Chemoattractant Protein-1 recruits monocytes, NK cells, memory T cells and dendritic cells. | Innate and adaptive | ✓ | ✓ |
| MIP-1α/CCL3 | Macrophage Inflammatory Proteins are responsible for chemotaxis of monocytes, T cells, NK cells and dendritic cells and have proinflammatory role. | Innate and adaptive |  | ✓ |
| MIP-1β/CCL4 |  |  |  | ✓ |
| RANTES/CCL5 | Regulated on Activation, Normal T cell Expressed and Secreted chemokine recruits T cells, eosinophils and basophils. | Innate and adaptive | ✓ | ✓ |
| Eotaxin/CCL11 | Eosinophil chemotactic protein. | Innate and adaptive |  | ✓ |
| MCP-5/CCL12 | Monocyte Chemotactic Protein 5 attracts eosinophils, monocytes and lymphocytes. | Innate and adaptive | ✓ | ✓ |
| TARC/CCL17 | Thymus- and Activation-Regulated Chemokine is produced by monocytes, macrophages and dendritic cells. It plays a role in helper T cells and T regulatory cell chemotaxis. | Innate and adaptive |  | ✓ |
| sICAM-1 | Soluble InterCellular Adhesion Molecule-1 is stimulated by cytokine like TNF-α and is involved in endothelial transmigration of leukocytes. | Innate |  | ✓ |
| TIMP-1 | Tissue Inhibitor of MetalloProteinase 1 inhibits matrix metalloproteinases and maintains the extracellular matrix composition. | Innate and adaptive | ✓ | ✓ |
| TREM-1 | Triggered Receptor Expressed on Myeloid cells 1 is associated with elevated pro-inflammatory cytokine production on stimulation of toll-like receptors. | Innate |  | ✓ |
| G-CSF | Granulocyte Colony Stimulating Factor stimulates bone marrow to produce granulocytes and stem cells to release them in blood stream. | Innate | ✓ | ✓ |
| M-CSF | Macrophage Colony Stimulating Factor stimulates bone marrow to produce macrophages and is involved in their proliferation and survival. | Innate |  | ✓ |
| GM-CSF | Granulocyte-Macrophage Colony Stimulating Factor promotes granulocyte and monocytes production from stem cell. | Innate | ✓ | ✓ |
| C5/C5a | Complement component 5a is an anaphylatoxin and is also responsible for recruitment of phagocytes and complement factors at the site of infection. | Innate |  | ✓ |
| **CYTOKINES** | | | | |
| IFN-γ | Interferon gamma helps protect against viral, bacterial and protozoan infection. | Innate and adaptive | ✓ | ✓ |
| TNF-α | Tumor Necrosis Factor alpha is involved in inflammation regulation and is produced by macrophages upon infection. | Innate | ✓ | ✓ |
| IL-1α | Interleukin-1 alpha is produced by neutrophils and macrophages. It is responsible for inflammation and fever. | Innate |  | ✓ |
| IL-1β | Interleukin-1 beta is a cytokine that regulates immune cells proliferation, differentiation and apoptosis. | Innate | ✓ | ✓ |
| IL-1ra | Interleukin -1 receptor antagonist is an inhibitor of IL-1α and IL-1β. | Innate |  | ✓ |
| IL-2 | Interleukin -2 stimulates growth of T cells. | Adaptive | ✓ | ✓ |
| IL-3 | Interleukin -3 works together with IL-5 and GM-CSF to ward off the bacteria by inducing an inflammatory response. It is produced by T cells and is involved in myeloid cell proliferation. | Innate and adaptive |  | ✓ |
| IL-4 | Interleukin -4 stimulates proliferation of activated B cells and T cells. | Adaptive | ✓ | ✓ |
| IL-5 | Interleukin -5 acts as eosinophil chemoattractant, increases secretion of IgA and is a stimulant for B-cells. | Adaptive |  | ✓ |
| IL-6 | Interleukin -6 acts as both pro-inflammatory cytokine and anti-inflammatory monokine, which helps in neutrophil production, B cell growth and is antagonistic to regulatory T cells. | Innate and adaptive | ✓ | ✓ |
| IL-7 | Interleukin -7 is involved in T and B cell development as it stimulates proliferation of lymphoid cells. | Adaptive |  | ✓ |
| IL-10 | Interleukin -10 act as an anti-inflammatory cytokine, it downregulates the expression of Th1 cytokines, MHC-II and costimulatory molecules on macrophage. It enhances B cell proliferation and function. | Innate and adaptive | ✓ | ✓ |
| IL-12p70 | Interleukin -12 p70 is produced by innate immune cells involved in T cell differentiation. | Innate and adaptive | ✓ | ✓ |
| IL-13 | Interleukin -13 is an anti-inflammatory cytokine, it induces matrix metalloproteinases (MMPs) and also induces secretion of IgE antibody. | Adaptive |  | ✓ |
| IL-16 | Interleukin -16 is a chemoattractant for activated T cells, monocytes, dendritic cells and eosinophils. | Innate and adaptive | ✓ | ✓ |
| IL-17a | Interleukin -17a is a proinflammatory cytokine produced by activated T cells. | Innate and adaptive | ✓ | ✓ |
| IL-18 | Interleukin -18 is produced mainly by macrophages. It helps in cell mediated immunity and regulates IFN-γ production. | Innate and adaptive | ✓ |  |
| IL-23 | Interleukin -23 acts on the function of other cytokines like IL-17, IL-6, TGF-β and promotes IFN-γ production. | Innate and adaptive | ✓ | ✓ |
| IL-27 | Interleukin -27 is involved in differentiation of T cell subsets. | Adaptive |  | ✓ |
| **IMMUNE CELL PHENOTYPES** | | | | |
| Myeloid cell | Myeloid cells are derived from bone marrow and comprise different red blood cells and granulocytes. They are mainly involved in innate immune response. | Innate |  |  |
| Monocyte | Monocytes are involved in antigen-presentation, phagocytosis and responsible for production of cytokines. They can differentiate into macrophages and dendritic cells. | Innate and adaptive |  |  |
| Monocyte-Macrophage | Monocyte-macrophages are differentiated macrophage from monocytes circulating in the blood. They are highly inflammatory and protective against infection. During inflammation they are recruited to the site of inflammation. | Innate and adaptive |  |  |
| Resident Macrophage | Resident macrophages in many organs are seeded during embryonic development and self-renew independently of blood monocytes. During inflammation, those tissue macrophages are accompanied and sometimes replaced by recruited monocyte-derived macrophages. | Innate and adaptive |  |  |
| Neutrophil | Neutrophils have phagocytic ability, anti-microbial effect and are the first cells that reach the site of inflammation. | Innate |  |  |
| Dendritic cell | Dendritic cells are a link between innate and adaptive immune system and are mainly known for antigen presentation to T-cells. | Innate and adaptive |  |  |
| Natural Killer cells | Natural Killer cells carry out their function either by directly killing the infected cell or by secreting cytokines to fight against the infection. | Innate and adaptive |  |  |
| B-cell | B-cells are involved in antibody production, cytokine secretion and antigen presentation. | Adaptive |  |  |
| T-cell | T-cells differentiate into subsets like helper T cells which are responsible for cytokine production, regulatory T cells that keep the immune response under check, cytotoxic T cells which are responsible for killing infected cells and memory T cells that help eradicate infection by retaining memory of the invading agent. | Adaptive |  |  |

**Figure S1.**


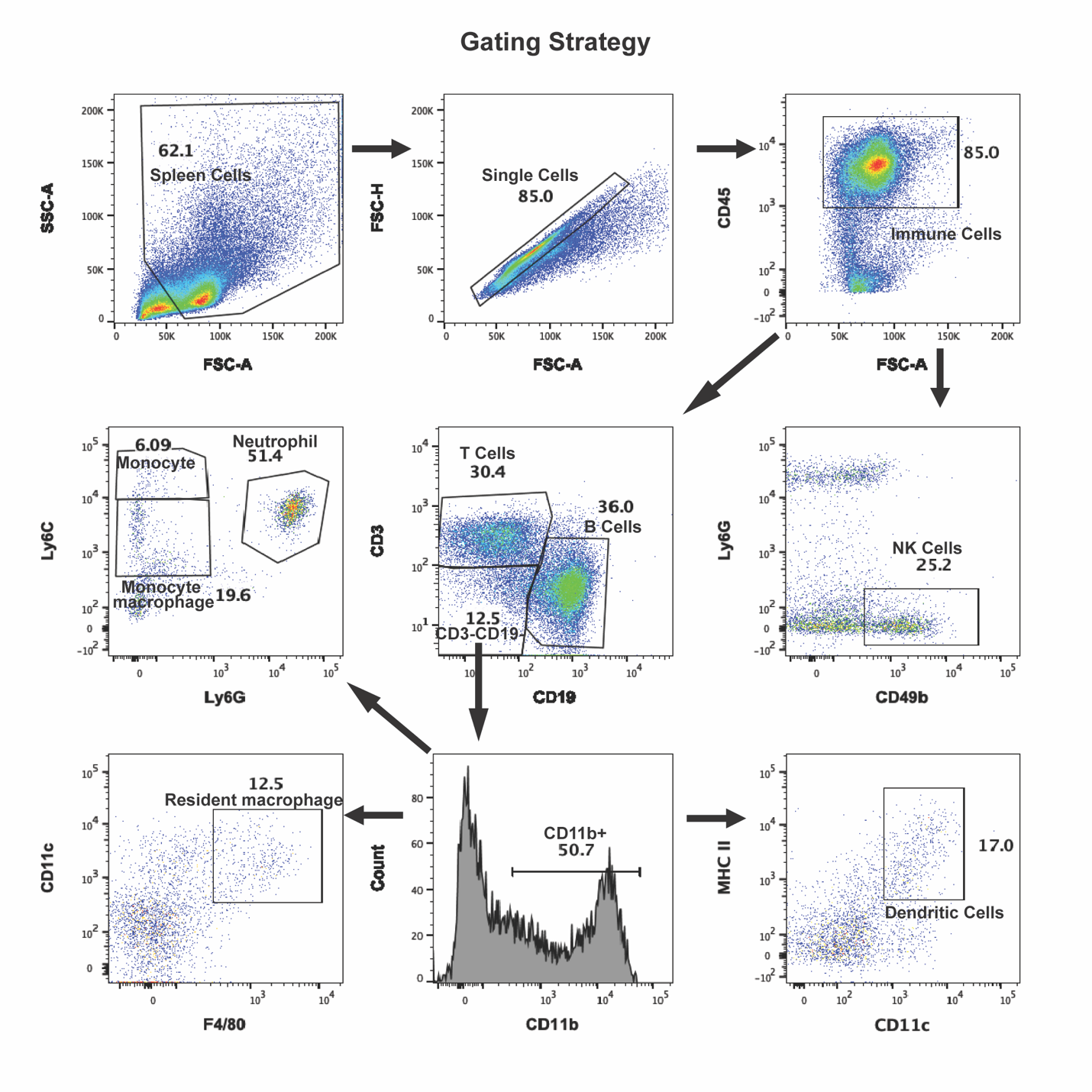


**Figure S1. Strategy applied to flow cytometry analysis** representing gating of spleen cells with different immune cell markers. Black arrow indicates gating from the parent population. X and Y axis are labelled with the specific fluorochrome markers used in the study.

**Figure S2.**


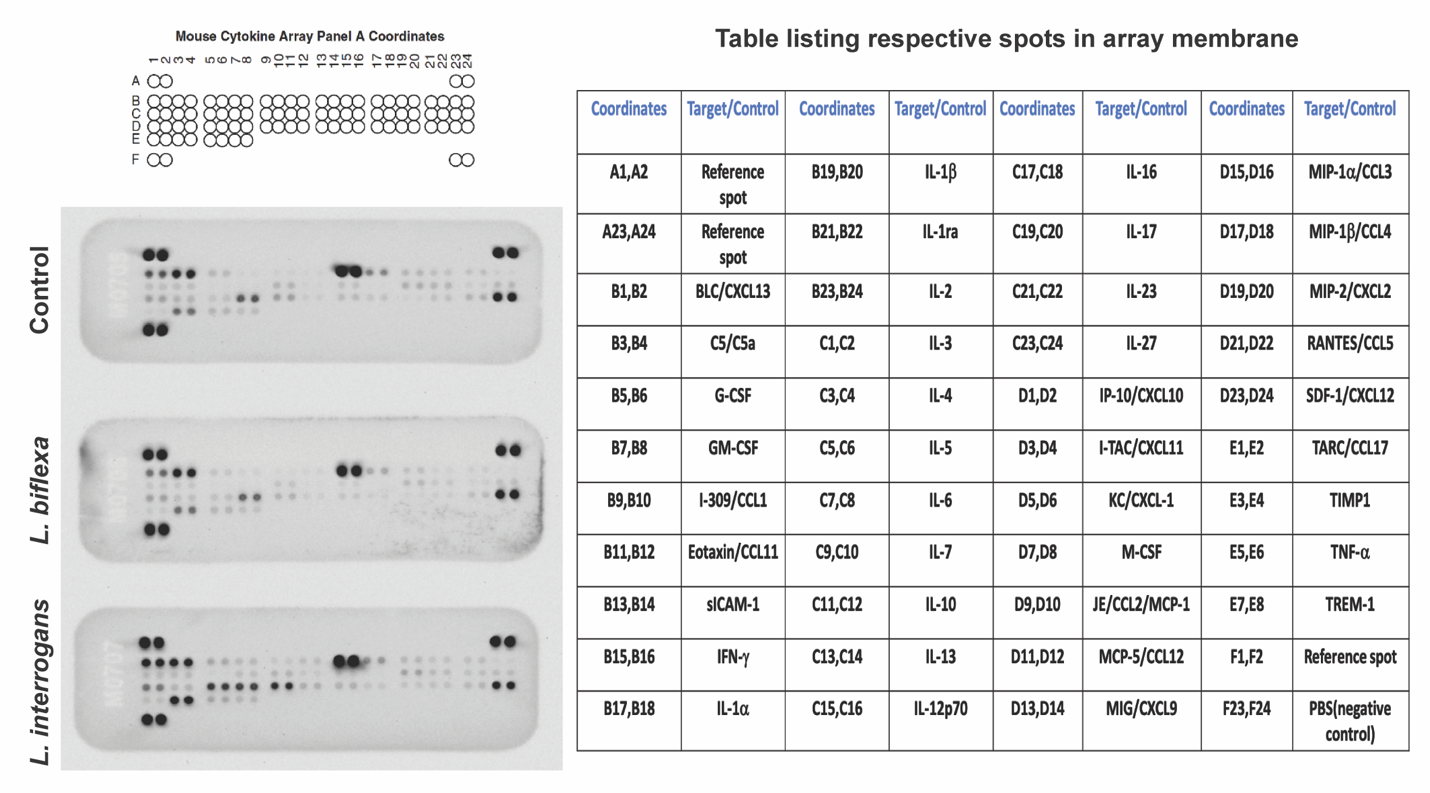


**Figure S2. Mouse proteome profile array coordinates representing cytokine and chemokine targets.** Upper left panel represents array membrane template with coordinates (spots). The table represents the reference spot (positive and negative control) and respective cytokine and/or chemokine corresponding to each coordinate. Lower left panel depicts examples of array membranes for different groups at 72h post infection (10 min exposure). Mean pixel density was analyzed after subtracting the background and normalized with each reference spot. Three different exposure times were considered to analyze the mean pixel density at 24h and 72h pi for all groups.

**Figure S3.**


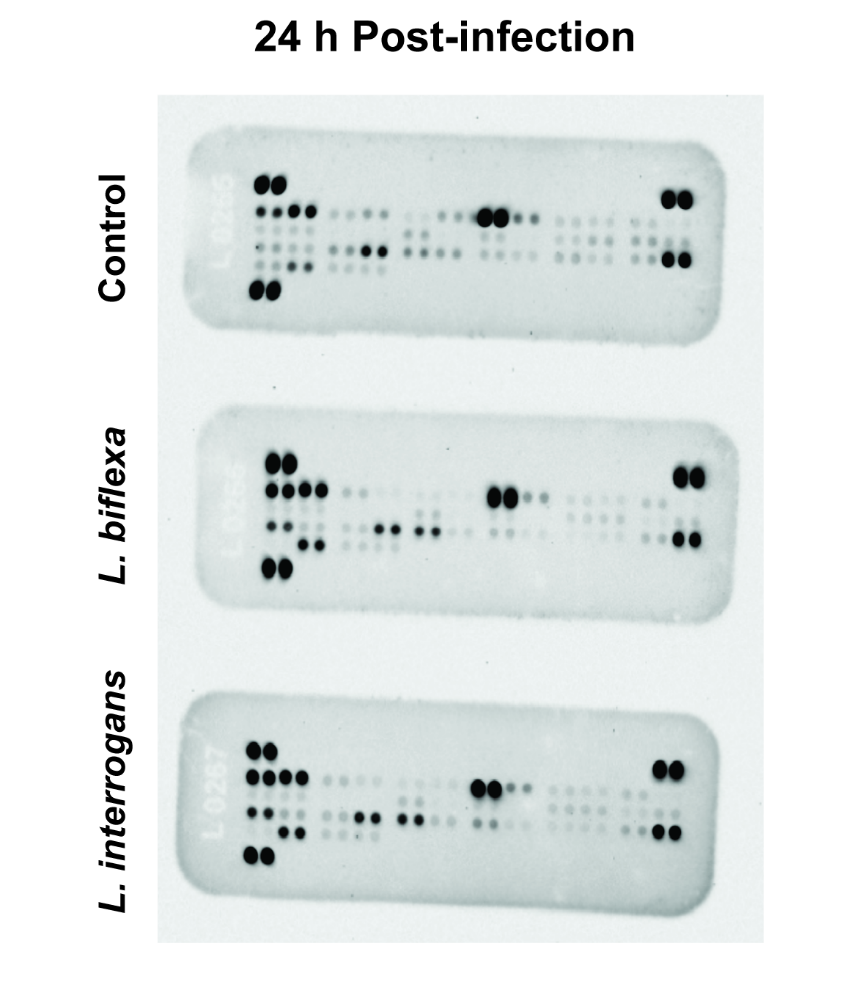


**Figure S3. Mouse proteome profile array membrane at 24h post infection.** Representative proteome profile array membrane for different groups at 24h post infection timepoint after 15 min exposure.
